# Controlled generation of high-intensity optical rogue waves by induced modulation instability

**DOI:** 10.1038/srep39926

**Published:** 2017-01-04

**Authors:** Saili Zhao, Hua Yang, Nengsong Chen, Chujun Zhao

**Affiliations:** 1 Key laboratory for Micro/Nano Optoelectronic Devices of Ministry of Education, College of Computer Science and Electronic Engineering, Hunan University, Changsha 410082, China; 2 College of Physics and Electronics, Hunan University, Changsha 410082, China

## Abstract

Optical rogue waves are featured as the generation of high amplitude events at low probability in optical systems. Moreover, the formation of optical rogue waves is unpredictable and transient in photonic crystal fibers. In this paper, we put forward a method to generate high-intensity optical rogue waves in a more controlled way based on induced modulation instability, which can suppress the noise effect and hence play a leading role in the process of pulse evolution. Our numerical simulations indicate that the generation of rogue wave can be controlled when seeding at the optimal modulation frequency and the intensity of rogue wave can be enhanced with appropriate modulation depth. Further, high-intensity rogue wave can also be ejected in the fiber with a shorter propagation length by regulating the modulation depth. These results all provide a better understanding of optical rogue wave, which can contribute to the generation of tunable long-wavelength spectral components and selective excitation of mid-infrared supercontinuum.

Optical rogue wave (RW) generated in fiber systems is a special wave, whose amplitude is significantly larger than that of the average wave. Another important characteristic of RW is the unpredictability in the sense that such wave seems to “appear from nowhere and disappear without a trace”^1^. The term RW originates from oceanography, which is mysterious and severely destructive in the ocean^2^,^3^,^4^. So far, the characteristic of oceanic RW has been found in different physical contexts, such as plasma waves^5^, Bose-Einstein condensations^6^, and nonlinear optics^7^. Optical RW, which is firstly discovered by Solli *et al*. in the photonic crystal fiber (PCF), is deemed to be an optical analogue of oceanic RW because of two reasons^8^. Firstly, the highly skewed L-shaped probability soliton intensity distribution with long tail can be regarded as a specified feature of RW, which predicts the occurrence of high amplitude event with a low probability^9^,^10^. Secondly, modulation instability (MI) can make optical RW generated when considering the initial noise perturbation, which is also a dramatic nonlinear effect related to the generation of oceanic RW^11^,^12^. Later, optical RW has also been found in many other optical systems, such as mode-locked laser^13^ and fiber Raman amplifiers^14^. Besides, the unusually steep and solitary profile of optical RW suggests a relevance to the soliton - broadband, solitary wave that can propagate without spreading on account of a balance between linear and nonlinear effects^8^,^15^. Therefore, optical RW is also called rogue soliton in the process of supercontinuum (SC) generation occurring in optical fibers^15^. The emergence of RW seems to be spontaneous, which is a sudden and rare event^16^,^17^,^18^. Nevertheless, the unpredictability and randomness of RW generation can affect the property of fiber systems, which do harm to the SC generation process. In order to make use of optical RW, how to manipulate the corresponding complex nonlinear dynamic behavior is becoming a hot topic.

As is well known, the RW formation is related to MI, which makes the small amplitude wave grow into a higher amplitude one^19^,^20^. Optical RW concept came from the studies of noise-induced soliton wavelength jitter during SC generation in fibers, where noise-induced fluctuations can modify the dynamic so as to generate rare-but-extreme event^21^,^22^. At the same time, RW generation is also influenced by other factors. For example, Dudley *et al*. demonstrated that RW generation can be enhanced by an order of magnitude through a small modulation across the input pulse envelope and suppressed through the use of a sliding frequency filter^7^; Buccoliero *et al*. showed that the RW would not occur in any SC spectrum limited by the fiber loss^23^. These conclusions manifest that the RW can be affected by changing incident conditions. When long pulse transmits in optical fiber, MI is responsible for the broadband noise amplification through phase-matched four-wave mixing (FWM), which leads to the ejection of a series of fundamental solitons and dispersive waves (DWs)^24^,^25^,^26^. Subsequently, because of Raman effect, multiple collisions between solitons or solitons and DWs cause the energy redistribution of these solitons and thus ultimately generate the RW^27^,^28^. While, previous work mainly focuses on how to predict and avoid the generation of RW in order to improve SC smoothness^8^,^23^. There are few papers exploring how to generate high-intensity RW in a more controlled manner. In 2010, K. Hammani *et al*. explored the emergence of RW events from turbulent fluctuations by the generalized nonlinear Schrödinger equation (GNLSE)^29^. Three turbulent regimes are identified that lead to the emergence of specific RW, namely, persistent and coherent rogue quasi-solitons, intermittent-like rogue quasi-solitons and sporadic RW events. The first regime is characterized by a robust and persistent coherent rogue quasi-soliton in the midst of small-scale fluctuation, which has been studied in various circumstances^30^,^31^. These phenomena show that even though the peak power of RW is changed, the RW can still maintain the quasi-soliton structure within quite some transmission distance. Moreover, A. Mathis *et al*. proposed a flexible means of studying the RW statistics, and examined the particular conditions leading to the extended tails in the associated probability distribution for the optical intensity^4^. The above work provides the possibility for the generation of high-intensity RW. Recently, H. Yang *et al*. demonstrated that RW can be generated by induced MI in the process of SC generation regardless of the noise effect^32^. However, noise is non-ignorable when long pulse propagates in optical fiber, which can exhibit distinct intensity fluctuations and have a significant influence on the initiation of the MI process. More importantly, the noise properties can be modified by induced MI so that the behavior of RW is changed to some extent. Actually, D. M. Nguyen *et al*. have reported the phenomenon that a low amplitude seed makes a great difference on the bandwidth and noise properties of picoseconds MI in a highly nonlinear fiber^33^. Therefore, in this paper, we focus on the propagation state of RW generated by induced MI when considering the noise effect. When the noise-induced MI dominates the pulse evolution, the generation of RW is random. However, because the induced MI can suppress the noise, the seed-induced MI can result in the controlled generation of RW at the optimal modulation frequency. Furthermore, the intensity of RW is enhanced by regulating the modulation depth. As a consequence of induced MI, controlled generation of high-intensity RW is obtained under the effect of the noise. Such RW can be used to obtain tunable spectrum components and selectively excite the SC. In addition, high power spectrum can be expanded to the mid-infrared wavelength by manipulating the generation of high-intensity RW. Thus, it is of great significance to investigate the controlled generation of high-intensity RW by induced MI.

The rest of this paper is organized as follows. MI gain spectral characteristic with high-order dispersion and Raman effects is depicted firstly. Based on the MI gain curve, the generation of RW under the varied modulation frequencies and depths is then discussed in detail. Furthermore, high-intensity RW can be obtained by changing the fiber length when adjusting the modulation depth. At last, we present the GNLSE model, which is the main equation governing the RW evolution in nonlinear medium^13^,^34^,^35^,^36^.

## Results

**Figure 1:**
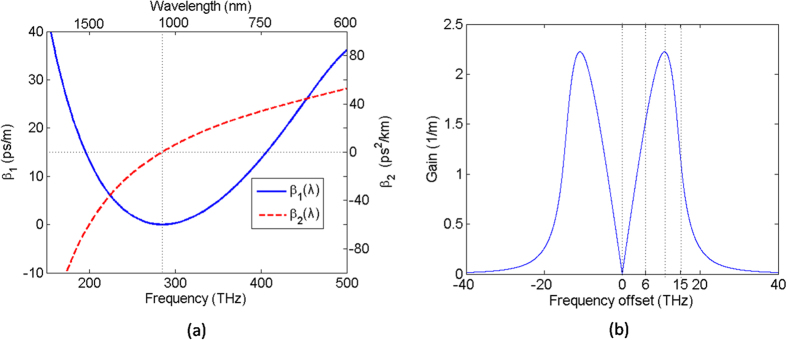
(**a**) The group delay *β*_1_ = *β*′(*ω*) (blue solid curve) and the related group-velocity dispersion *β*_2_ = *β*″(*ω*) (red dash curve) as the function of the frequency and wavelength. The dotted line is located the zero dispersion wavelength at the 1055 nm. (**b**) MI gain spectrum (blue solid curve) for the pump power of 150 W as the function of seed frequency offset. The dotted lines are located at the frequency offset of 0 THz, 6 THz, 11.75 THz and 15 THz.

The behavior of RW depends on the incident parameters to a great extent, which makes it possible to manipulate the RW generation by changing the initial conditions. Meanwhile, PCF is selected as a suitable transmission medium due to its controllable dispersion and nonlinear characteristics^37^. Dispersion exists in essentially all wave supporting physical systems, which typically exhibits a dependence of propagation speed on wave vector. So, an exemplary group delay profile *β*_1_ = *β*′(*ω*) and related group-velocity dispersion profile *β*_2_ = *β*″(*ω*) are depicted in Fig. 1(a), where *β*(*ω*)as the propagation constant is expanded in a Taylor series around the carrier frequency *ω*_0_. The related dispersion parameters of the PCF are from the ref. 38. Figure 1(b) shows the range of MI gain spectrum where the frequency offset is positive corresponding to seeding at a wavelength shorter than that of the pump. The effect of high-order dispersion up to the tenth order and Raman scattering effect are included to obtain MI gain spectrum, which is calculated for the pump peak power alone.

For the controlled generation of high-intensity RW, we numerically simulate the propagation of the pump and the seed launched into the anomalous dispersion regime of the PCF with 30 m length. In this section, we select different modulation frequencies and depths to regulate the RW generation, where the induced MI plays an important role in the process of pulse evolution. Moreover, the intensity plots use the same logarithmic density scale in order to better analyze the pulse evolution with different modulation frequencies and depths.

**Figure 2:**
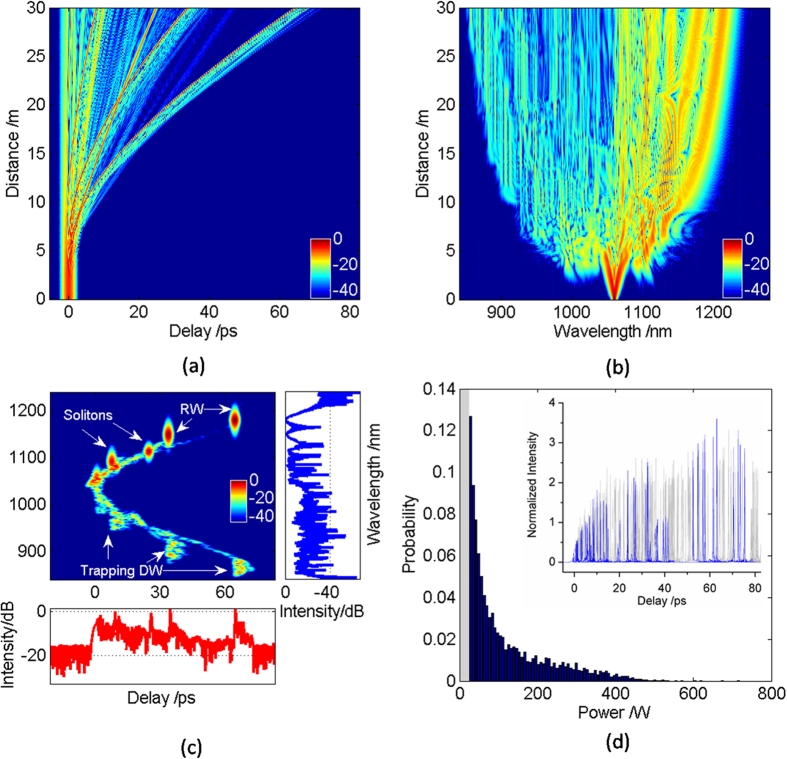
Density plots of (**a**) temporal evolution and (**b**) spectral evolution pumped at 1060 nm under the unseeded condition. (**c**) The related output spectrogram. (**d**) The intensity distribution histogram of 100 simulations, which presents a close-up on low power events. The inset shows the corresponding output pulse shapes of 100 simulations, where the lines with blue and gray color indicate output pulse shapes with and without RW generation, respectively.

Figure 2 illustrates the RW generation with zero modulation frequency in the PCF, namely, unseeded RW generation. Figure 2(a) shows the typical temporal evolution of soliton fission, where a series of fundamental solitons are ejected after a few meters of propagation distance. These fundamental solitons are with different durations and different peak powers, and thus multiple collisions occur between them because of different group velocities. What’s more, multiple collisions between solitons have been suggested as a possible driver mechanism behind RW. At the same time, the interaction between the DWs and fundamental solitons is also an important mechanism for the RW generation. Owing to Raman effect, the RW has a larger time delay in the time domain compared with other fundamental solitons. The corresponding RW can also be demonstrated in the spectral evolution in Fig. 2(b), which locates at the longest wavelength. When only the pump wave propagates in the PCF, MI can lead to spontaneous breakup of the sub-picosecond beam into a periodic pulse train. In this case, noise acts as a probe and is amplified by the gain provided by MI. Moreover, noise can cause significant SC fluctuations and induce bifurcation-like divergence of different pulse trajectories, which indicates that the noise is favor of RW generation to some extent. In order to better observe the RW, the output spectrogram is used to describe the spectral and temporal contents of the signal simultaneously in Fig. 2(c). Under the unseeded condition, MI grows from the noise. Subsequently, the noise-induced MI leads to high-order soliton fission, which breaks up into a number of solitons that interact and transfer energy between each other during multiple collisions. The interaction will add to the noise, which depends largely on the related phases and amplitudes of solitons. While, there is still a prior transfer of energy from the smaller solitons to the larger ones. The energy transfer can lead to the formation of RW, whose amplitude is two times higher than that of the average wave height^1^,^2^,^8^,^10^. Simultaneously, the RW has a bigger time delay and larger red-shift compared with fundamental solitons. Moreover, when the group-velocity matching condition between the red-shift RW and DW is satisfied, the RW can capture the DW, which contributes to the generation of new frequency components^39^,^40^.

Considering the randomness of noise, output pulse shapes of 100 simulations are presented under the different noise seeds in Fig. 2(d), which is statistical appearance of RW. The line with blue color indicates the output pulse shape with RW generation, where the amplitude of RW is two times higher than that of the surrounding average wave and the corresponding time delay is also larger than that of other waves. While, that line with gray color indicates the output pulse shape without RW generation. In the unseeded case, the RW is only generated in a few numerical simulations by the noise-induced MI, which is uncontrollable and has a larger difference in the time delay. To further analyze the statistical properties of RW, the related intensity distribution histogram of 100 simulations is also showed in Fig. 2(d). More importantly, the histogram presents a close-up on low power events so that a clear comparison can be observed in the statistic of RWs. We can see that the intensity distribution histogram exhibits the ‘L-shaped’ long-tailed characteristic of extreme value optical RW events. Similar result has been found from previous studies^9^,^10^, which illustrated that the emergence of RW is high amplitude event with a low probability under the unseeded condition. Thus, the generation of RW is extremely unstable resulting from the effect of noise-induced MI. In order to obtain a more controlled generation of RW by reducing the noise effect, numerical simulations are presented with different modulation frequencies in the following figures.

**Figure 3:**
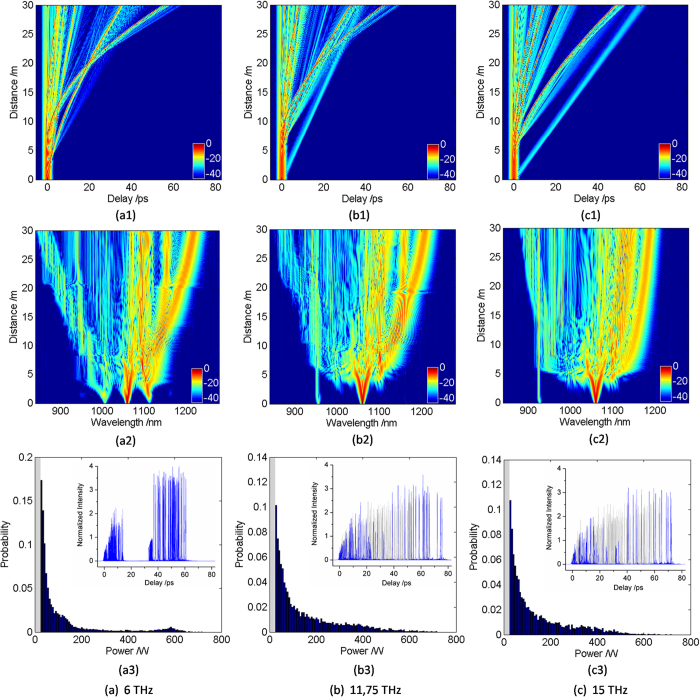
(1) Temporal evolution (upper row), (2) spectral evolution (middle row) and (3) The intensity distribution histogram of 100 simulations (lower row) pumped at 1060 nm with a seed of 0.1 modulation depth at the modulation frequency of (**a**) 6 THz, (**b**) 11.75 THz, (**c**) 15 THz, respectively. These histograms all present a close-up on low power events. In addition, the inset (lower row) shows the corresponding output pulse shapes of 100 simulations, where the lines with blue and gray color indicate output pulse shapes with and without RW generation, respectively.

To reduce the noise effect, the seed with a frequency offset relative to the pump is provided within the MI gain spectrum. Figure 3 shows the RW generation with different modulation frequencies (6 THz, 11.75 THz, 15 THz), which demonstrates the temporal and spectral evolution of pulse envelope at the same modulation depth *a*_0_ = 0.1. We find that, because of the interaction between anomalous dispersion and nonlinear effects, MI leads to the fission of input pulse into a number of fundamental solitons after the initial broadening stage caused by self-phase modulation (SPM). Next, due to the high-order dispersion and Raman Scattering perturbation, fundamental solitons have different group velocities, which cause the collisions between solitons. As mentioned above, the RW, which has an extreme red-shift in the temporal domain, can be generated by multiple collisions between solitons or solitons and DW radiations. Simultaneously, the corresponding RW can also be found at the longest wavelength in the spectral evolution.

FWM is one of the most fundamental processes in nonlinear optics when long pulse as the pump. The key properties of FWM can be observed in the degenerate case in which two pump waves are at the same frequency, and the nonlinear interaction involves the conversion of the pump into a pair of parametric sidebands. With any initial seed, FWM corresponds to the instability of the propagating long pump and the growth from noise of sidebands symmetric in frequency about the pump^26^. For a 6 THz modulation frequency, the breakup of the input pulse is initiated by the FWM process, which has been investigated in refs 32, 41 and 42. Interestingly, MI gain spectrum is the degenerate FWM gain spectrum of the pump, which amplifies the FWM cascade of the pump and the seed. Therefore, a single set of sidebands can be amplified by the degenerate FWM in the process of spectral evolution. The second set of sidebands are shifted 16 THz away from the pump, which just locate at the position with smaller MI gain and therefore can quickly move outside of the gain spectrum when the pump depletes. At the propagation distance of about 5 m, a series of fundamental solitons are ejected from the long wavelength sideband. Since the peak powers and durations of solitons are different, the corresponding red-shift rates change. Shortly afterwards, multiple collisions occur between solitons, which lead to the energy exchange of solitons. Overall, multiple collisions make the energy preferentially transferred from the smaller soliton to the larger one and thus ultimately generates the RW, which undergoes the largest Raman induced frequency shift (RIFS). It should also be noted that the RW located at the longest wavelength is formed from one of FWM sidebands at a very short propagation distance of about 7 m. For a 11.75 THz modulation frequency, which is at the peak of MI gain, decreased set of sideband is slowly amplified by FWM process compared with the situation as shown in Fig. 3(a2). The second sideband is shifted 31 THz away from the pump, whose intensity is very weak due to the corresponding weak MI gain spectrum. Besides, due to the effect of noise, the second sideband only locates at the shorter wavelength than that of the pump. With the depletion of the pump, MI gain spectrum shifts to the shorter wavelength, which leads to the SC narrower compared with Fig. 3(a2). And seeding at the peak of the MI gain spectrum can also bring about the RW generation. For a 15 THz modulation frequency, the collision doesn’t occur between fundamental solitons in the temporal evolution in Fig. 3(c1), which is related without the formation of RW in Fig. 3(c2). In this case, the seed is shifted to the tail of the MI gain spectrum and thereby the generated FWM is very weak and asymmetrical. Even though the additional signal power is increased to the pump by the temporally overlapping, the induced MI gain doesn’t have much effect on the process of RW generation. The case is similar to that unseeded, where the RW is formed at a small probability.

In order to better elaborate the probability of RW generation under different modulation frequencies, the corresponding output pulse shapes of 100 simulations are presented in the bottom figures of Fig. 3. These lines with gray color indicate the output pulse shapes without RW generation, while those with blue color indicate ones with RW generation. By contrast, we find that the RW has a larger and more stable amplitude as well as more intensive time delay under the condition of 6 THz. However, for the modulation frequency 11.75 THz or15 THz, the generation of RW, a small probability event, can’t be effectively controlled. Meanwhile, the RWs have a lower intensity and more dispersed time delay compared with Fig. 3(a3). This phenomenon can be explained by the fact that the MI gain spectrum is strongly influenced by the Raman effect. The seed with 6 THz modulation frequency is much closer to the peak of the Raman gain in contrast to that with 11.75 THz or 15 THz. On this occasion, the spectral evolution is dominated by the amplification of a set of coherent sidebands, which leads to the appearance of more collisions between solitons or solitons and DWs. Thus the probability of RW generation can be enhanced when the seed is placed at 6 THz. Especially, the similar case has also been proposed in ref. 41, where a seed at approximately the Raman gain peak, namely three-fifths of the MI gain peak, gives an optimum improvement of the RW stability. To show the statistical properties of RW with different modulation frequencies in more detail, the corresponding intensity distribution histograms of 100 simulations are presented in Fig. 3(a3–c3). We can observe that these histograms all present the ‘L-shaped’ long-tailed characteristic. Moreover, by comparing the probabilities at the position of extremely high power, we find some intriguing phenomena. When the modulation frequency is 11.75 THz or 15 THz, the statistical probability of RW emergence is slightly higher than that of the unseeded situation. Nevertheless, the generation of RW is still a small probability event in Fig. 3(b3) and (c3). Interestingly, the histogram has a relatively large raise in the vicinity of 580 W when the modulation frequency is 6 THz. Obviously, the raise indicates that the probability of RW emergence is much stronger in contrast with other cases. In addition, the tail of intensity distribution histogram is relatively stable under the condition of 6 THz, which shows that RW can be generated in a more controllable manner. As a result, under the influence of noise, the generation of tunable RW can be controlled at the optimal modulation frequency which is determined by the initial MI-Raman gain. What’s more, the RW redshift contributes to the further broadening at the expense of SC smoothness.

**Figure 4:**
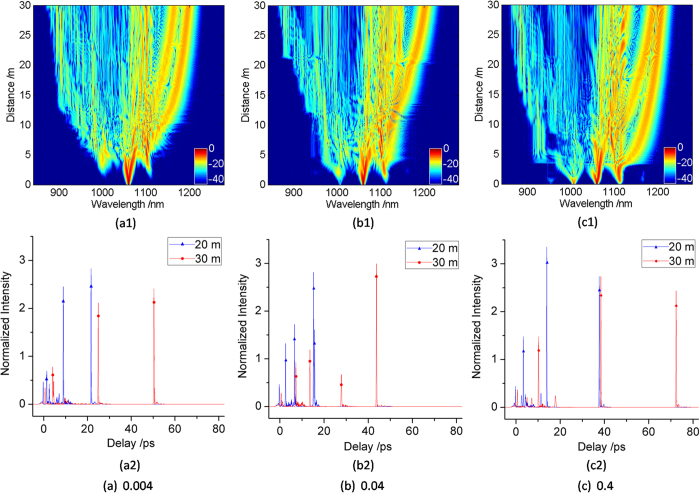
(1) The spectral evolution (upper row) pumped at 1060 nm with a seed at the 6 THz modulation frequency for different modulation depths ((**a**) 0.004, (**b**) 0.04, (**c**)0.4), respectively. (2) The corresponding pulse shapes with RW generation (lower row) for the propagation distance of 20 m (blue line with triangle) and 30 m (red line with round).

For a thorough understanding of the induced MI dynamics, let us further consider the RW generation over a range of modulation depths, as shown in Fig. 4. The upper figures show the spectral evolution for different modulation depths (0.004, 0.04, 0.4) at the modulation frequency of 6 THz. The bottom figures show the related pulse shapes with RW generation at the propagation distance of 20 m and 30 m, respectively. For modulation depth 0.004, the formation of effectively FWM sidebands needs a comparatively long propagation distance due to the weak value of the seed. As the modulation depth gradually increases, more FWM sidebands located at the two sides of the pump are generated at a shorter propagation distance. The above phenomena imply that the deeper the modulation depth, the stronger the fluctuation on the pump, which is beneficial to the formation of more FWM sidebands. With the increase of propagation distance, the RWs emerge at about 8 m, 11 m, and 3.5 m in the spectral evolution at the modulation depth of 0.004, 0.04, and 0.4, respectively. By comparing among the upper figures of Fig. 4, it is found that RW can be generated in a shorter propagation distance under either very weak (Fig. 4(a1)) or large (Fig. 4(c1)) modulation depth. For very large modulation depth, the input pulse power is high, which leads to the decrease of MI period. The soliton can obtain enough red-shift to facilitate the RW generation at the relatively short propagation distance. For very weak modulation depth, even though the soliton undergoes insufficient Raman shift, the RW can be ejected by absorbing enough energy because of frequent soliton collisions. In addition, the phenomena of Raman shift are also observed in the bottom figures, which demonstrate that the RWs have different Raman shifts under the different modulation depths when propagating at the same distance. By comparing these pulse shapes at the propagation distance of 30 m, the RW in Fig. 4(b2) has the smallest Raman shift. Besides, with the increase of propagation distance from 20 m to 30 m, the intensity of RW is enhanced in Fig. 4(b2) while decreased in Fig. 4(a2) and (c2). The reason is as follows. At the initial stage of RW formation, the intensity of RW is growing rapidly by constantly absorbing other waves’ energy. To a certain degree, the RW stops absorbing the energy and hence the intensity of RW is decreased, which has also been observed in ref. 43. Under the condition of different modulation depths, the positions of RW ejected-firstly are different. With the increase of propagation distance, the energy of RW is gradually increased due to the energy transfer. When the propagation distance reaches a certain degree, the RW has the highest intensity. Therefore, high-intensity RW can also be obtained when the RW propagates at the optimal fiber length. These features show that high-intensity RW can be generated by choosing suitable modulation depth and fiber length.

**Figure 5:**
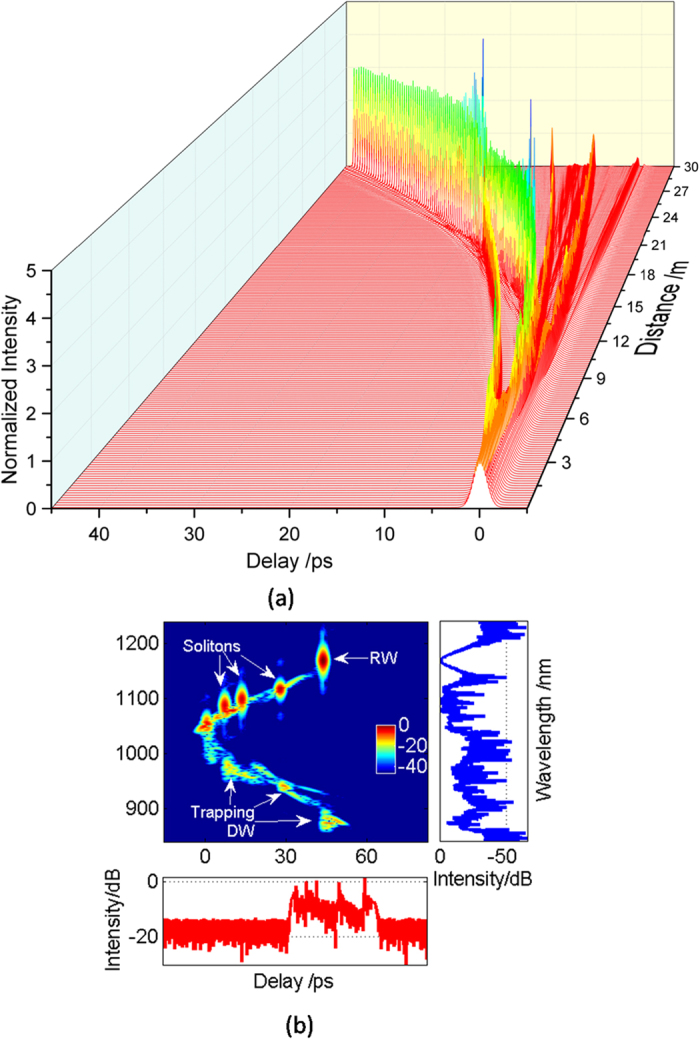
(**a**) Three-dimensional temporal evolution along the fiber pumped at 1060 nm with a seed of 0.04 modulation depth at the 6 THz modulation frequency. (**b**) The corresponding output spectrogram.

In order to obtain a more intuitive image of the RW generation, three-dimensional temporal evolution and the corresponding output spectrogram are demonstrated in Fig. 5, which is under the same condition with Fig. 4(b). Compared with the steady propagation at the distance less than 3 m, the output RW has a 45 ps deviation from the initial linear trajectory in the time domain, which is attributed to the Raman shift. At the same time, the temporal shift is accompanied by the change of pulse energy. In the initial stage, there are two fundamental solitons with relatively low intensity generated in the PCF, as indicated in Fig. 5(a). The soliton at the shorter wavelength transfers energy to the other soliton at the longer wavelength, which induces a subsequent change in the velocity, namely, the increase of Raman shifting rate. When the propagation distance increases to 14 m, the RW with high intensity is generated due to multiple collisions between solitons or solitons and DWs. It is obvious that the collisions take place at 14 m, and thereby the solitons get reshaped with the change of peak energy. The output spectrogram also shows that the RW has a higher intensity and a larger red-shift compared with other fundamental solitons in Fig. 5(b). When the solitons are split from the pump in the anomalous dispersion regime, the DWs are simultaneously excited from the pump in the normal dispersion regime. When the group-velocity matching condition between the red-shift soliton and DWs is satisfied, the soliton can trap the DWs. Moreover, the RW has a higher intensity compared with that in Fig. 2(b). Therefore, high-intensity RW can be generated in a more controlled way by induced MI.

## Discussion

In this paper, we have discussed a fascinating mechanism that contributes to the controlled generation of high-intensity optical rogue wave in the photonic crystal fiber. The mechanism shows that the behavior of rogue wave can be significantly modified by changing the incident parameters such as modulation depth and modulation frequency. In addition, by selecting the optimal incident parameters, high-intensity rogue wave can be obtained within a relatively short propagation length based on the induced modulation instability. The rogue wave generated in a more controllable manner can guide tailoring the supercontinuum and generating new specific frequency components. Therefore, we believe that this paper provides a new insight in the controlled generation of high-intensity rogue wave in a multitude of physical scenarios.

## Methods

It has been well known that GNLSE, taking group velocity dispersion (GVD), SPM, stimulate Raman scattering effects into account, suffices to observe the RW in the process of SC generation. The GNLSE can be expressed as ref. 37:


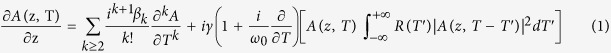


where *A*(*z, T*) represents the pulse envelope, *T* denotes the time in a reference frame moving at the group velocity of the input pulse, *z* accounts for longitudinal coordinate along the fiber axis, *β*_*k*_ is the *k*th-order dispersion coefficient at the central frequency *ω*_0_, and *γ* is the nonlinear coefficient. *R*(*t*) = (1 − *f*_*R*_)*σ*(*t*) + *f*_*R*_*h*_*R*_(*t*) is the nonlinear response function, which includes both instantaneous electronic and delayed Raman contributions, with *f*_*R*_ = 0.18 representing the Raman contribution and *h*_*R*_ determined from the experimental fused-silica Raman cross-section.

The RW dynamics are typically observed when SC is generated by induced MI at the initial stage, and thereby the sub-picosecond excitation is considered under the similar condition^41^,^44^. In this paper, we model a Gaussian pump pulse (pulse width *T*_0_ = 500 fs and center wavelength *λ*_0_ = 1060 nm) and seed pulse propagating in the fiber. The modulated Gaussian input pulse envelope can be expressed as:


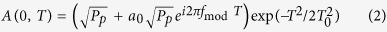


where *P*_p_ = 150 W is the peak power of the pump, and the power of the seed is defined as 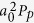, *a*_0_ is the modulation depth. The seed pulse is temporally overlapped with the pump, and gives a frequency offset *f*_mod_. In addition, the noise needs to be considered when the sub-picosecond pulse propagates in the optical system^21^,^45^. Although it is random and has a lower power, the noise can affect the temporal and spectral evolution when amplified by MI. The split-step Fourier method is adopted to solve the GNLSE.

## Additional Information

**How to cite this article**: Zhao, S. *et al*. Controlled generation of high-intensity optical rogue waves by induced modulation instability. *Sci. Rep.* **7**, 39926; doi: 10.1038/srep39926 (2017).

**Publisher's note:** Springer Nature remains neutral with regard to jurisdictional claims in published maps and institutional affiliations.
